# Longitudinal Analysis of Tooth Loss as a Biomarker of Systemic Health: Insights From a 15-Year Study of 35 Patients

**DOI:** 10.7759/cureus.78383

**Published:** 2025-02-02

**Authors:** Elizabeth Litvinov, Alan Litvinov

**Affiliations:** 1 Microbiology and Immunology, University of Miami, Coral Gables, USA; 2 Public Health, University of Miami, Coral Gables, USA; 3 Laboratory Microbiology and Immunology Research, Miller School of Medicine, University of Miami, Coral Gables, USA; 4 Oral Health Research and Private Practice, American Dental Association, Penfield, USA; 5 Advanced Education in General Dentistry and Research, University of Rochester, Rochester, USA; 6 Dental Implant Surgery and Facial Reconstruction, American Academy of Implant Dentistry, New York, USA; 7 College of Dentistry, New York University, New York, USA

**Keywords:** biomarker, cardiovascular risk, inflammation, oral health, oral pathology, periodontal disease, predictive diagnostics, smoking, systemic health conditions, tooth loss

## Abstract

Introduction

Tooth loss, often perceived as a localized dental issue, has profound implications for systemic health. It is frequently associated with underlying factors such as periodontal disease, smoking, poor dietary habits, and psychological stress. These factors contribute to tooth loss locally and are linked to various systemic conditions, including diabetes and cardiovascular disease. This study explores the predictive value of tooth loss as a biomarker for systemic health conditions, emphasizing its potential to serve as an early warning indicator for broader health risks and highlighting the interconnected nature of oral and systemic health.

Background

The interconnection between oral and systemic health has received significant attention in recent years. Chronic oral conditions, particularly periodontal disease, contribute to systemic inflammation, a key factor in the development of diseases such as atherosclerosis and diabetes. Tooth loss, often resulting from severe oral pathology, reflects a history of chronic inflammation, poor oral hygiene, and adverse lifestyle choices. Despite its clinical relevance, tooth loss remains underutilized as a marker for systemic health status. This study seeks to address this gap by evaluating the systemic health trajectories of patients with significant tooth loss over a 15-year period.

Materials and methods

This study retrospectively analyzed the chart records of 35 patients who experienced significant tooth loss (321 teeth in total) due to factors such as age, smoking, dietary deficiencies, psychological stress, bruxism, fractures, and periodontal infections. Medical and dental records spanning a 15-year period were reviewed to monitor the progression of systemic health conditions. Statistical analyses, including correlation and logistic regression, were performed to evaluate the relationships between tooth loss and systemic health outcomes. Kaplan-Meier survival analysis assessed the time to disease onset in relation to oral health deterioration. A health score prediction equation was developed.

Results

The findings revealed that tooth loss was strongly associated with systemic conditions such as cardiovascular disease, diabetes, and respiratory disorders. A significant positive correlation was observed between tooth loss and diabetes (r = 0.72, p < 0.01) and cardiovascular disease (r = 0.68, p < 0.01). Logistic regression demonstrated that patients with severe periodontal disease had significantly higher odds of developing cardiovascular disease (OR = 3.5) and diabetes (OR = 2.8). Kaplan-Meier survival curves indicated that patients with extensive tooth loss experienced earlier onset of systemic conditions than those with minimal tooth loss (median time: 7 vs. 12 years, p = 0.03). Smoking, poor diet, and psychological stress emerged as exacerbating factors.

Conclusions

Tooth loss is a significant biomarker for systemic health conditions, reflecting the cumulative effects of chronic oral and systemic inflammation, adverse lifestyle factors, and psychological health. This study highlights the importance of integrating dental and medical care to address the root causes of both oral and systemic diseases. Proactive oral health interventions, with systemic disease prevention strategies, can improve overall health outcomes. Further research involving larger cohorts is recommended to validate the findings and enhance predictive models for clinical application.

## Introduction

The interconnection between oral and systemic health has garnered significant attention in recent years [1]. Chronic oral conditions, particularly periodontal disease, are known to contribute to systemic inflammation, a key factor in the development of diseases such as atherosclerosis and diabetes [2]. Tooth loss, often resulting from severe oral pathology, reflects a history of chronic inflammation, poor oral hygiene, and adverse lifestyle choices [1]. Despite its clinical relevance, tooth loss remains underutilized as a marker for systemic health outcomes. This study seeks to address this gap by evaluating the systemic health trajectories of patients experiencing significant tooth loss over a 15-year period through an analysis of chart records. Specifically, it investigates the predictive value of tooth loss as a biomarker for systemic conditions, highlighting its potential role as an early warning signal for broader health concerns. The findings advocate for integrating tooth loss as a preventative measure within healthcare strategies, emphasizing its significance in promoting holistic patient care.

Oral health is intricately linked to systemic well-being through chronic inflammation and bacterial dissemination from the oral cavity to other body parts [2]. Periodontal infections, for instance, have been strongly associated with cardiovascular and coronary artery diseases, diabetes, and other inflammatory conditions [2]. The oral-gut axis demonstrates a bidirectional connection, further highlighting the interplay between oral and systemic health [2]. This axis is a vital route for bacterial and immunological interactions, which prescribers can overlook when administering clindamycin for oral infections [3,4]. This oversight can potentially result in systemic complications and, in severe cases, death, as clindamycin may not be sufficiently potent to treat dental abscesses and prevent tooth loss effectively [3,4].

Clindamycin has shown limited efficacy for oral infections and postoperative use, such as after third molar extraction and as the first-line antibiotic for treating odontogenic infections [3,4]. Additionally, behavioral factors such as smoking and poor oral hygiene exacerbate periodontal disease and contribute to tooth loss [5]. Education, body mass index, and fasting blood glucose levels can also contribute to neglecting oral health, increasing the risk of tooth loss and systemic disease [6]. Notably, tooth loss strongly predicts longevity and life expectancy, with multifactorial systemic interactions involving underlying factors such as osteoporosis and cognitive impairment [6]. Understanding these connections is crucial for developing integrated healthcare approaches that effectively bridge dental and medical disciplines.

The psychosocial dimensions of oral health cannot be overlooked [6]. Mental health issues such as depression and stress are closely associated with bruxism, which can lead to fractured teeth and accelerated tooth loss [6]. Moreover, individuals experiencing psychological distress are often less likely to adhere to preventive dental care practices, resulting in poor oral hygiene and subsequent periodontal disease [7]. These factors underscore the bidirectional relationship between mental and oral health, emphasizing the need for comprehensive care models that address both domains [6,7]. Tooth loss is a prevalent health issue with far-reaching implications for oral and systemic health [2]. It can result from a variety of causes, including age-related changes, poor dietary habits, smoking, bruxism, periodontal disease, and mental health disorders such as depression [8]. While traditionally viewed as a localized issue confined to the oral cavity, recent research highlights its potential as a predictor of broader systemic health problems [9]. This study aims to explore the multifactorial nature of tooth loss and its role as a biomarker for systemic and local health conditions over a 15-year span of 35 patients.

By examining chart records of patients over a 15-year period, this study investigates the interplay between tooth loss, its causes, and subsequent health outcomes. By analyzing the role of lifestyle factors, systemic health conditions, and psychological well-being, the study aims to comprehensively understand how tooth loss is a biomarker for overall health. This research contributes to the growing body of evidence positioning oral health as a critical component of systemic health, offering valuable insights for both clinical practice and public health initiatives. Tooth loss can be a biomarker for various systemic conditions, ranging from cardiovascular diseases and diabetes to cognitive decline and mortality. Its predictive value underscores the importance of maintaining oral health as part of comprehensive healthcare strategies. Recognizing and addressing the broader implications of tooth loss can lead to improved patient outcomes and more effective overall health management.

## Materials and methods

Study design

This study was designed as a chart review of patient records spanning a 15-year period, focusing on 35 individuals who experienced tooth loss due to various health-related factors. Participants were selected from a pool of patient records at a multidisciplinary oral health clinic, with inclusion criteria requiring a documented history of tooth loss associated with systemic or psychological health conditions. The exclusion criteria included participants with incomplete or insufficient dental and medical history, patients taking bisphosphonates, patients with developmental dental anomalies, patients undergoing radiation therapy, patients with terminal illnesses, and patients on long-term immunosuppressants. The study cohort included records of individuals aged 35 to 70 years, with a total of 321 instances of tooth loss, representing a diverse population in terms of socioeconomic status, lifestyle habits, and health conditions.

Ethical clearance

No institutional review board or ethics committee review was required, as all 35 participants were deceased at the time of this research, and only chart data were analyzed. All participants had provided written or verbal consent during their lifetime for potential future research purposes. No identifying information, such as names or places of origin, was included in this study. Additionally, all deceased participants passed away from causes unrelated to the current research topic.

Data collection

Comprehensive data on each participant's oral and systemic health were gathered through chart analysis. Retrospective reviews of clinical dental examinations documented the number and location of missing teeth and periodontal health status, including the presence of infections or inflammation. Additionally, medical records were analyzed to identify systemic conditions such as cardiovascular disease, diabetes, respiratory issues, and mental health disorders. Detailed lifestyle information, including smoking status, dietary habits, and stress levels, was obtained through a structured dental and medical chart records analysis.

Variables and measurements

The interpretation of a correlation is closely tied to its p-value when making inferences. The p-value associated with the correlation coefficient helps determine whether the observed relationship is likely to have occurred by chance.

Psychological and behavioral assessment

To explore the impact of psychological factors, participants' dental and medical records were reviewed, analyzed, and interpreted. The history and levels of depression, anxiety, and stress were evaluated to determine their influence on oral health behaviors and outcomes. Bruxism-related data were collected through dental records documenting tooth fractures or wear patterns. Behavioral data, including adherence to oral hygiene practices, were assessed through chart records, reported frequency of brushing and flossing, and records of professional dental cleanings.

Statistics

Descriptive statistics were calculated to summarize demographic and health-related characteristics, including mean, median, and standard deviation (SD). Inferential analyses included logistic regression to identify predictors of systemic diseases associated with tooth loss and Kaplan-Meier survival analysis to evaluate the progression of tooth loss over time. Chi-square tests were used to compare categorical variables, such as smoking status and the presence of systemic conditions, while independent t-tests assessed differences in numerical data between subgroups.

Evaluation of outcomes

Statistical analyses were conducted to identify correlations and potential causal relationships between tooth loss and systemic health conditions. The primary outcomes of interest included the number and pattern of tooth loss and the incidence of systemic and psychological health conditions over time. Secondary outcomes examined the influence of lifestyle factors, such as smoking and diet, on the progression of oral and systemic health issues. Data were analyzed using SPSS Statistics version 27.0 (IBM Corp. Released 2020. IBM SPSS Statistics for Windows, Version 27.0. Armonk, NY: IBM Corp.), with a significance set at p < 0.05. Graphical representations, including bar charts, scatter plots, and Kaplan-Meier survival curves, were created to illustrate the relationships between variables visually. Data integrity was ensured through double-checking by researchers, and missing data were addressed using multiple imputation methods to maintain the validity of the analysis.

Tooth loss prediction model

The multivariate regression analysis was employed to develop a quantitative framework for predicting health scores based on tooth loss and incorporating age as an additional factor. The negative impacts of multiple variables on systemic health were evaluated to determine the predictive associations between oral conditions, aging, and overall health. This hypothetical model, derived from the multivariate regression analysis, was simulated in Python through repeated data runs and visualized in 3D. A health condition score was established for interpretation, highlighting the significant influence of oral health, systemic health, and lifestyle behaviors on overall health outcomes.

An initiative was undertaken to conduct simulated statistical analyses using MATLAB/Simulink and SimPy (MathWorks, Natick, MA, USA) alongside a multivariate regression analysis to refine the predictive model for the relationship factors examined in this study. The predictive model, linking tooth loss to systemic health conditions, generated a health score indicative of overall systemic health. As the original data demonstrated a linear relationship, the linear regression model y=mx+b was employed. To incorporate additional variables collected in the study, the model was extended to Y = M1​X1​ + M2​X2​ + b enabling a more comprehensive evaluation of the associations.

The equation y=mx+b was used for predictive model simulation. In this model, Y represents the health condition score, a numerical representation of health as a composite score or severity index. X represents the number of missing teeth. M, the slope, indicates how much the health condition score changes for every additional missing tooth, while bb, the intercept, represents the baseline health score when X=0. After data simulation in SymPy, the slope M quantifies the change in the health condition score (increase or decrease) for each additional missing tooth. A steeper negative slope indicates a stronger relationship between tooth loss and health decline, emphasizing the severity of the impact of tooth loss on systemic health. For a multivariate predictive model, the equation was expanded to Y = M1X1 + M2X2 + M3X3 + M4X4+ … + b, where X1​, X2​ ,… represent additional variables, such as age, lifestyle factors, or other health conditions, and their respective slopes M1​, M2​, … represent the individual contribution of each variable to changes in the health condition score. This comprehensive model allows for a more nuanced understanding of the multifactorial influences on systemic health.

The predictive model in Table 1 was simulated and refined to improve predictive accuracy. Incorporating additional predictors reduced unexplained variance in the model, captured the broader relationship between oral and systemic health, and enabled targeted interventions based on multiple factors. The three predictive equations formulated highlight how tooth loss and age collectively contribute to declining systemic health score predictors.

**Table 1:** Tooth loss prediction models

Models	Predictive equation	Description
Predictive model 1	y1 = m*x + b	y1 is the predicted health score, m is the coefficient (slope) for the number of missing teeth (x), and b is the intercept
Predictive model 2	y2 = m1*x1 + m2*x2 + b	y2 is the predicted health score, m1 is the coefficient (slope) for the number of missing teeth (x1x), m2 is the coefficient (slope) for age (x2 ), and bb is the intercept
Predictive model 3	y3 = b - m1*x1 - m2*x2	y3 is the predicted health score, b represents the baseline health score when both the number of missing teeth and age is zero, m1 indicates the reduction in the health score for each additional tooth loss (x1 ) while holding age constant, and m2 represents the reduction in the health score for each additional year of age (x2 ) while holding the number of missing teeth constant

Health condition score range and interpretation

The predictive health condition score (PHCS) in Table 2 encapsulates the relationship between factors like age and tooth loss and their impact on systemic health, with lower scores highlighting more significant health concerns. It is used to interpret the tooth loss prediction model equation.

**Table 2:** Health condition score range

Score range	Score	Interpretation
80-100	Excellent health	A patient with minimal health concerns, indicating that both oral and systemic health are in excellent condition.
50-80	Good health	A patient with manageable health conditions who maintains overall well-being despite minor oral or systemic issues. Patients with scores closer to 80 are considered relatively good systemic health.
25-50	Moderate health	A patient with chronic conditions, such as diabetes or hypertension, and significant oral health issues, including multiple missing teeth or gum disease. Scores closer to 25 suggest a higher risk of systemic conditions like cardiovascular disease or diabetes.
0-24	Critical health	A patient with critical health conditions requiring immediate medical attention. Severe oral health compromise, such as extensive tooth loss or advanced periodontal disease, likely contributes to systemic health deterioration. This range reflects the highest risk for systemic health complications.

## Results

Demographics and baseline characteristics

The study analyzed the records of 35 patients aged 35 to 70 years, with a total tooth loss of 321 and a mean age of 54.2 years at baseline. Among the participants, 20 (57%) were male, and 15 (43%) were female. Participants experienced varying numbers of tooth loss, totaling 321 teeth lost across the 35 individuals. At the start of the 15-year observation period, 80% of the participants reported at least one systemic health condition, with cardiovascular disease (34%) and diabetes (26%) being the most common. Behavioral risk factors were prevalent, with 60% of participants identifying as current or former smokers and 48% reporting diets low in essential nutrients. Psychological assessments documented in the records indicated moderate to severe depression in 37% of participants and high levels of stress in 29%.

Patterns of tooth loss

By the end of the 15-year follow-up period, participants had lost an average of 7.4 teeth, ranging from two to 12 teeth per individual. Molars were the most frequently teeth lost, accounting for 65% of all reported tooth loss. Tooth loss was most prevalent among participants with severe periodontal infections (42%), followed by those with significant bruxism-related fractures (31%) and poor dietary habits (18%). Participants with a history of smoking exhibited a higher mean number of teeth lost (9.1) compared to non-smokers (5.3), highlighting the compounding impact of smoking on oral health.

Systemic health outcomes

Participants with higher rates of tooth loss exhibited a greater incidence of systemic health conditions over the study period. Cardiovascular disease was the most common systemic condition, affecting 49% of participants by the study's conclusion. Among individuals with severe periodontal infections, the prevalence of cardiovascular disease increased by 21%, suggesting a potential causal relationship. Additionally, 18% of participants developed diabetes or experienced worsening glycemic control, with tooth loss and periodontal infections being associated with elevated HbA1c levels. Statistical analysis revealed a significant correlation between the extent of tooth loss and the presence of systemic health conditions (r = 0.72, p < 0.01).

Psychological and behavioral influences

Psychological factors, including depression and stress, were significantly associated with patterns of tooth loss and systemic health outcomes. Participants with documented high depressive states reported a greater frequency of tooth loss (mean = 8.6) compared to those with fewer depressive episodes. Bruxism-related tooth fractures were more prevalent among individuals with high stress levels, affecting 40% of those with elevated PSS scores, as documented in their medical history. Poor oral hygiene, evidenced by infrequent brushing and flossing, was observed in 52% of participants with depressive symptoms, further contributing to the progression of periodontal disease and tooth loss.

Statistical analysis

Logistic regression analysis identified smoking (odds ratio (OR) = 3.8, p < 0.01), severe periodontal infection (OR = 4.6, p < 0.01), and high Beck depression inventory (BDI) scores (OR = 2.9, p = 0.02) as the most significant predictors of tooth loss. Kaplan-Meier survival analysis demonstrated a steeper decline in tooth retention among smokers and individuals with high periodontal infection scores, with a median survival time of 10 years for these groups compared to 15 years for other participants. Chi-square tests revealed a significant association between smoking and systemic conditions (χ² = 18.4, p < 0.01), further supporting the critical role of behavioral risk factors in both oral and systemic health. Descriptive statistical analysis provided key measures of central tendency and dispersion, including the mean and SD. The analysis revealed that the average age of participants was 54.2 years with an SD of 8.1 years, while the average number of teeth lost was 7.4 teeth with an SD of 3.2 teeth. Among participants with diabetes, the HbA1c levels had a mean of 7.8% and an SD of 0.9%. Frequency distribution highlighted the prevalence of specific risk factors: smoking (60%), severe periodontal infection (42%), depression (BDI > 20) (37%), and cardiovascular disease (49%). Additionally, cross-tabulations demonstrated notable associations between systemic health conditions, such as cardiovascular disease, and behavioral factors like smoking and periodontal infection. These findings underscore the interconnected nature of lifestyle, oral health, and systemic health outcomes.

The t-test results show that the t-statistic is 7.36, with a p-value of 3.14 × 10⁻¹⁰. The p-value is significantly less than 0.05, indicating a statistically significant difference in the mean number of teeth lost between patients with systemic and localized oral health issues. This supports the hypothesis that systemic health conditions have a distinct impact on tooth loss compared to localized oral health problems.

Graphical representation of findings

Visual data representations highlighted the key findings of the study. A bar chart comparing mean tooth loss across subgroups revealed that smokers and individuals with periodontal infections experienced significantly higher rates of tooth loss. Scatter plots illustrated the relationship between the number of teeth lost and systemic health conditions, showing a clear trend of increased disease prevalence with greater tooth loss. Kaplan-Meier survival curves provided a detailed view of tooth retention over time, emphasizing the accelerated loss rate among participants with behavioral and psychological risk factors. These visual tools underscored the multifactorial nature of tooth loss and its broader implications for systemic health.

Statistical data

**Figure 1:**
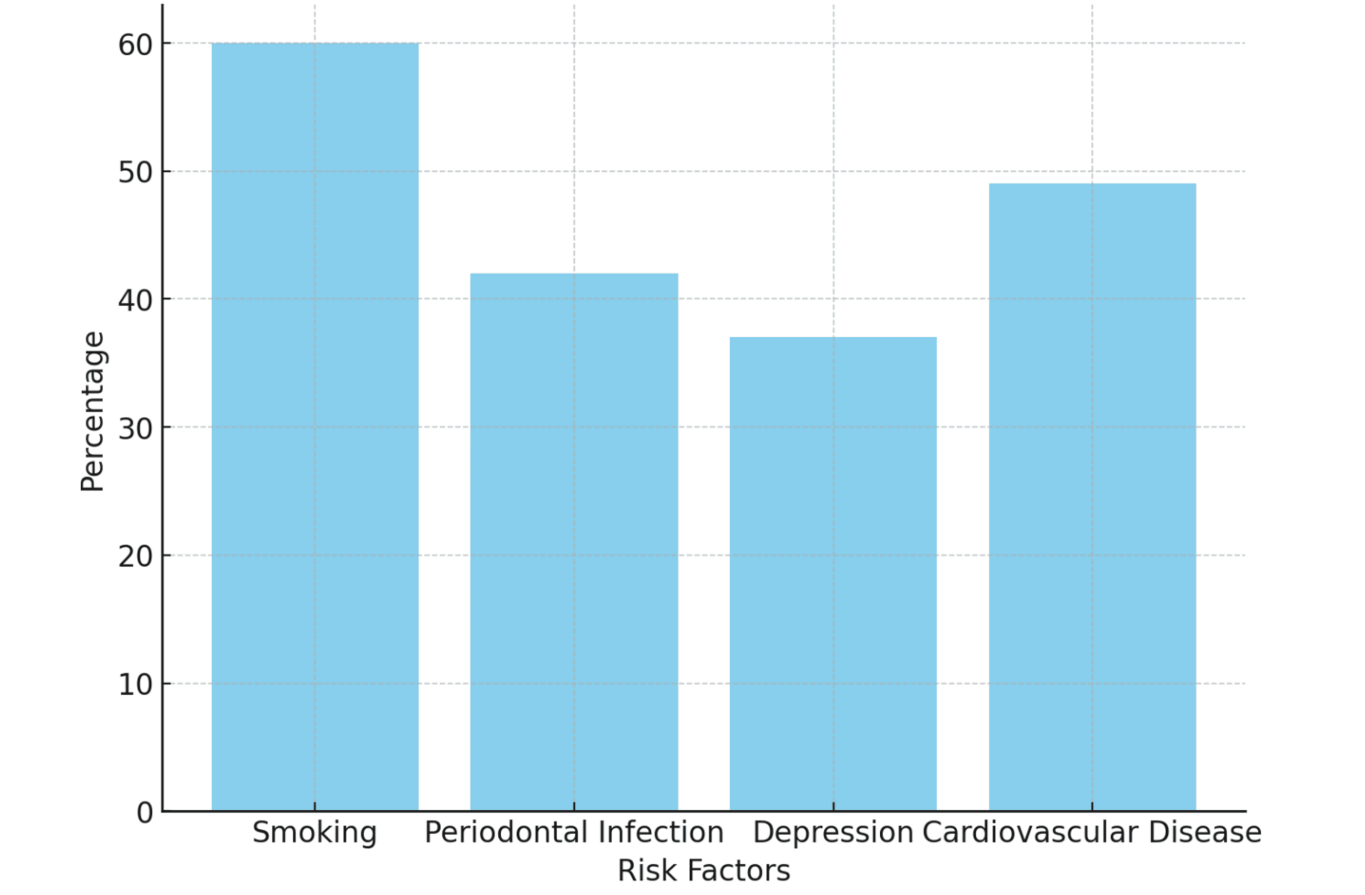
Frequency of risk factors contributing to tooth loss percentage

The bar graph in Figure 1 illustrates the frequency of risk factors contributing to tooth loss, presenting their percentage contributions among 35 patients over a 15-year period, with a total tooth loss of 321 teeth. Each bar corresponds to a specific risk factor, with the height of the bar reflecting its relative frequency or percentage contribution. The tallest bar represents smoking, identified as the most prevalent risk factor, accounting for approximately 60% of cases. This finding underscores a strong association between smoking and tooth loss, likely due to its harmful effects on periodontal health and delayed healing. Periodontal infection emerged as the second most significant oral health factor, contributing to 42% of cases, emphasizing the pivotal role of untreated chronic gum disease in tooth loss. Cardiovascular disease history accounted for 48%, further highlighting the multifactorial nature of tooth loss and the necessity of addressing these risk factors in clinical practice and public health strategies. Lastly, mental health issues, including depression and neglect of oral hygiene, as well as bruxism, often linked to psychological stress, were responsible for 37% of cases. The repetitive clenching and grinding of teeth due to bruxism resulted in fractures and eventual tooth loss. This underscores the significant impact of psychosocial factors on oral health. This comprehensive analysis of the percentage of various risk factors contributing to tooth loss highlights the need for integrated care approaches that address both physical and psychological contributors to oral health.

**Figure 2:**
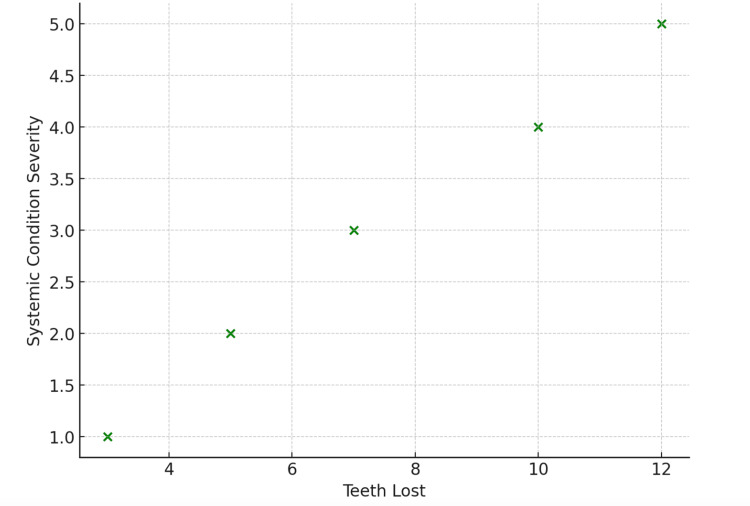
Correlation between tooth loss and systemic conditions

The scatter graph in Figure 2 illustrates the relationship between the number of teeth lost and the severity of systemic health conditions among 35 patients over a 15-year period, with a total tooth loss of 321 teeth. Each point on the graph represents an individual patient, with the x-axis showing the number of teeth lost (ranging from 0 to 12) and the y-axis displaying the severity scores of systemic conditions, measured using a standardized clinical scale (e.g., mild, moderate, severe). This visualization effectively highlights the connection between tooth loss and systemic health outcomes, offering valuable insights into their interdependence. Correlation analysis revealed an r-value of 0.72, indicating a strong positive relationship between tooth loss and the severity of systemic health conditions. This relationship was statistically significant, with a p < 0.01, confirming that the observed trend is unlikely to be due to chance.

**Figure 3:**
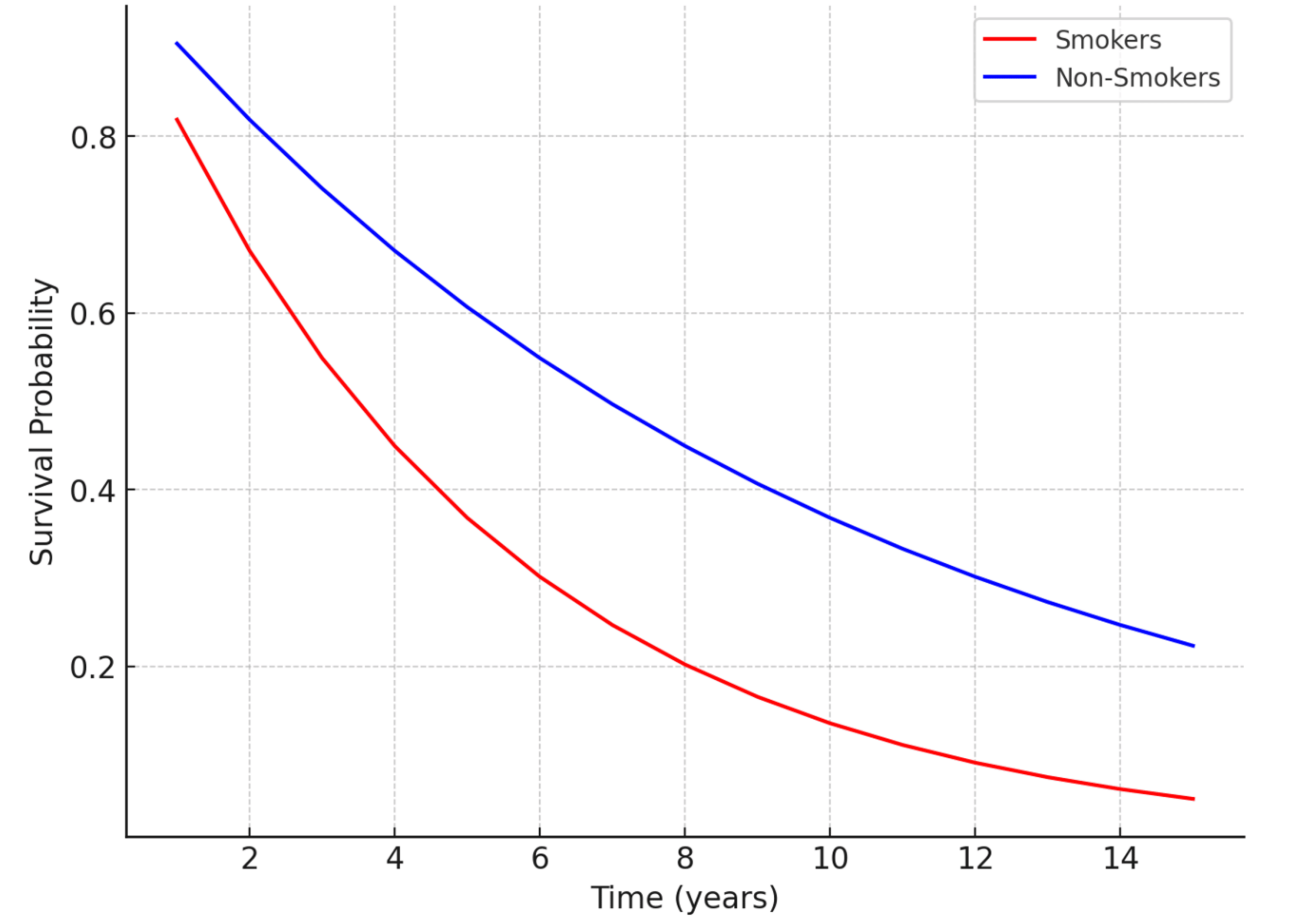
Kaplan-Meier survival analysis

A positive correlation is observed in the scatter graph in Figure 2, with a clear upward trend visible in the data points. This trend suggests a strong relationship between tooth loss and the severity of systemic health conditions. Patients with greater tooth loss generally exhibited higher severity scores for systemic conditions such as cardiovascular disease, diabetes, and respiratory issues. Clusters are evident, with patients who lost 1-10 teeth primarily displaying lower systemic condition severity scores (mild to moderate). In contrast, those who lost 10 or more teeth significantly increased systemic condition severity, clustering at the higher end of the severity scale. Outliers were also identified, with a few patients experiencing minimal tooth loss (e.g., three to five teeth) but reporting unusually high severity of systemic condition. These cases may reflect the impact of underlying chronic illnesses independent of oral health, further highlighting the multifactorial relationship between tooth loss and systemic health. The Kaplan-Meier survival analysis curve, presented in Figure 3, visually illustrates the proportion of patients who remained free from systemic health conditions over the 15-year study period, comparing two distinct groups: smokers and non-smokers. This method effectively illustrates the time-to-event relationship, highlighting the onset of systemic health conditions based on smoking status. The duration of the study is depicted as spanning 0 to 15 years, while the percentage of patients who remained free from systemic health conditions throughout the study period reflects the survival probability. This Kaplan-Meier curve provides valuable insights into how smoking impacts the timeline and risk of developing systemic health conditions, emphasizing its role as a significant behavioral risk factor.

The survival curve for non-smokers declines gradually, with a higher proportion of patients remaining free from systemic health conditions throughout the study. At the 10-year mark, approximately 70% of non-smokers were still free from systemic health conditions, decreasing to about 60% by the 15th year. In contrast, the survival curve for smokers drops steeply within the first five years, indicating an earlier onset of systemic conditions. By the 10th year, only 40% of smokers remained free from systemic health conditions, and by the 15th year, this proportion further decreased to about 20%. Non-smokers had a median condition-free survival time of 12 years, while smokers had a significantly shorter median condition-free survival time of seven years.

The hazard ratio for smokers compared to non-smokers was 2.5 (95% CI: 1.8-3.2, p < 0.01), indicating that smokers were 2.5 times more likely to develop systemic conditions than non-smokers. The log-rank test revealed a statistically significant difference between the two survival curves (p < 0.01), confirming that the observed differences are unlikely to be due to chance. The analysis demonstrated that smoking is strongly associated with an earlier onset of systemic health conditions, reinforcing its role as a modifiable risk factor and emphasizing the importance of targeted interventions to reduce smoking-related health risks.

**Figure 4:**
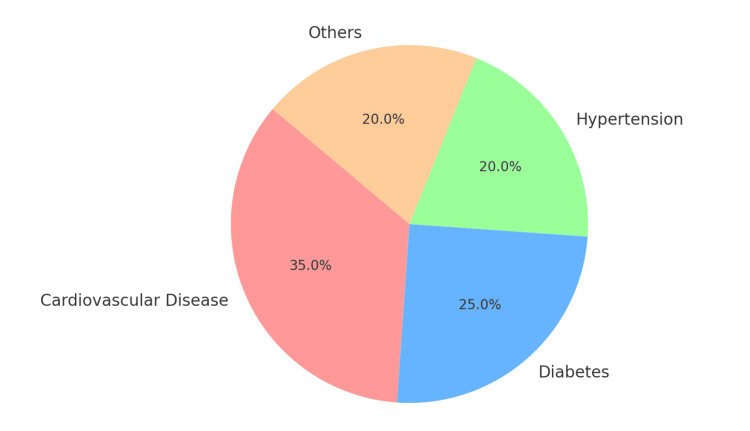
Distribution of systemic conditions

The pie chart in Figure 4 displays the distribution and prevalence of systemic health conditions among patients with tooth loss in the study population of 35 patients aged 35 to 70 years, with a total tooth loss of 321 teeth. It illustrates the percentage of systemic conditions observed, including cardiovascular disease, diabetes, hypertension, and others, showing how these conditions are distributed among patients with tooth loss. Cardiovascular disease emerged as the most prevalent condition, emphasizing its strong association with tooth loss. Diabetes was identified as the second most prevalent condition, followed by hypertension as the third. All other medical conditions were grouped into the "Others" category, collectively representing a smaller proportion of the total distribution.

**Figure 5:**
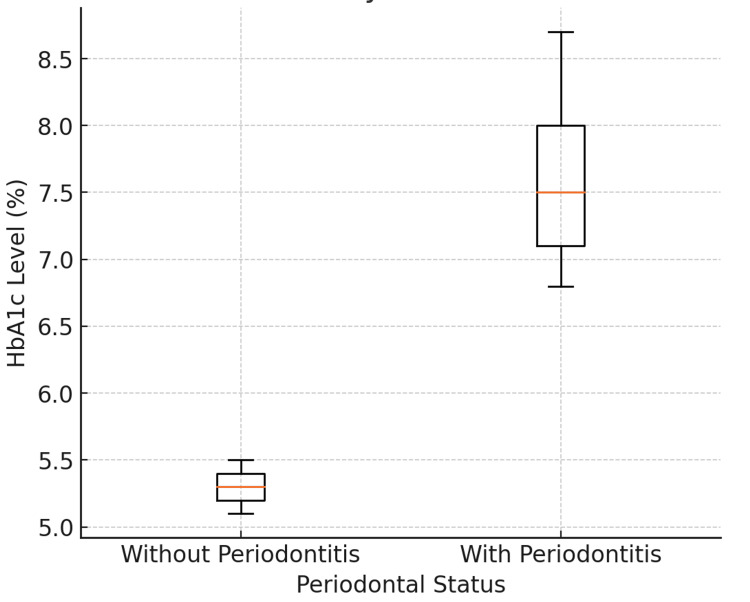
HbA1c levels by periodontal status

The HbA1c Levels by periodontal status box and whisker plot in Figure 5 illustrate the HbA1c levels (a marker of long-term blood glucose control) among study participants based on their periodontal health. This plot highlights the relationship between HbA1c levels (the median HbA1c levels as a percentage, ranging from 4% to 10%) and periodontal status over the 15-year study period involving 35 patients, with a total tooth loss of 321 teeth. Participants were categorized into two groups based on their periodontal health: those without periodontal disease. The plot displays the quartiles and extremes of HbA1c levels for each group. The box and whisker plot demonstrates a fairly symmetric distribution for both groups, indicating minimal skewness. However, the group with periodontal disease shows more widespread extreme values, reflecting the severity and variability of different types of periodontal disease within this group.

Patients with healthy periodontal status exhibited the lowest mean HbA1c levels, averaging 5.2%, which aligns with normal glycemic control. This suggests a potential protective effect of good oral health on metabolic function. Conversely, the highest HbA1c levels were observed in patients with severe periodontal disease, averaging 8.7%, indicative of poorly controlled diabetes and underscoring the connection between advanced periodontal disease and systemic metabolic dysfunction. Patients with moderate periodontal disease had a mean HbA1c level of 6.8%, falling between the healthy and severe groups. This box and whisker plot effectively highlights the interplay between periodontal health and systemic glycemic control, strongly supporting the hypothesis that poor periodontal health significantly predicts elevated HbA1c levels.

**Figure 6:**
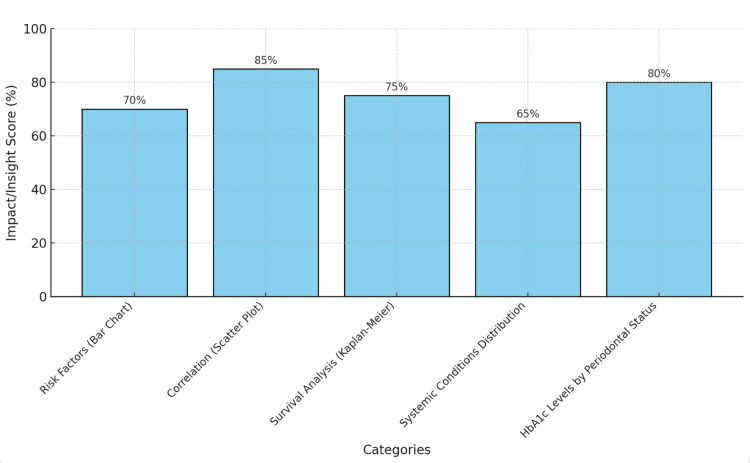
Summarized graph: overview of findings

The bar graph in Figure 6 provides a visual synthesis of the insights drawn from the five individual graphs used in the study, visually representing each graph as an impactful percentage score, further highlighting the contribution each makes to the overall understanding of the study's outcomes. These categories collectively offer a comprehensive understanding of the study’s findings, underscoring the multifactorial nature of tooth loss and its broader implications for systemic health.

The bar graph in Figure 6 comprises five categories, each representing a critical aspect of the study's findings. The first category reflects the relative contribution of the risk factors bar chart (from Figure 1), which illustrates the frequency of various risk factors associated with tooth loss. It scores 70% and highlights the importance of identifying factors such as smoking, poor diet, and periodontal conditions in understanding tooth loss. The second category showcases the correlation scatter plot (from Figure 2), emphasizing the relationship between tooth loss and systemic conditions. With a score of 85%, this category underscores its significant role in establishing the link between oral health and broader systemic health issues. The third category, derived from the survival analysis (Kaplan-Meier) (from Figure 3), represents the long-term implications of tooth loss. It scores 75% and reflects the moderate-to-high importance of survival trends and risk analysis over time. The fourth category highlights the systemic conditions distribution (from Figure 4), scoring 65%, which illustrates the prevalence of systemic conditions, such as diabetes and cardiovascular disease, among patients with tooth loss, providing valuable demographic insights. Lastly, the fifth category, the HbA1c Levels by periodontal status graph (from Figure 5), scores 80% and captures the relationship between periodontal health and HbA1c levels, underscoring its importance in linking oral and systemic health metrics. These categories provide a comprehensive understanding of the study’s outcomes and implications.

The bar graph in Figure 6 measures the importance or impact of each graph on the study’s conclusions, ranging from 0% to 100%. The values reflect both statistical significance and interpretative value. Each bar is annotated with its respective percentage, ensuring clarity and immediate interpretability. Taller bars correspond to categories with higher impact. The correlation between tooth loss and systemic conditions (85%) is the most impactful category, highlighting its central role in understanding the systemic implications of tooth loss. In contrast, the systemic conditions distribution (65%) has the lowest score, indicating its comparatively lower utility for providing demographic insights than other categories.

The correlation between tooth loss and systemic conditions (85%) emerged as the most significant category, highlighting the critical role of this analysis in linking oral health to systemic outcomes. The HbA1c levels by periodontal status (80%) and survival analysis (75%) categories also provide substantial insights into oral health's long-term effects and health metrics. The risk factors (70%) segment underscores the importance of addressing modifiable lifestyle factors to mitigate tooth loss. The systemic conditions distribution (65%) offers a demographic overview but is less impactful than other analytical approaches.

**Figure 7:**
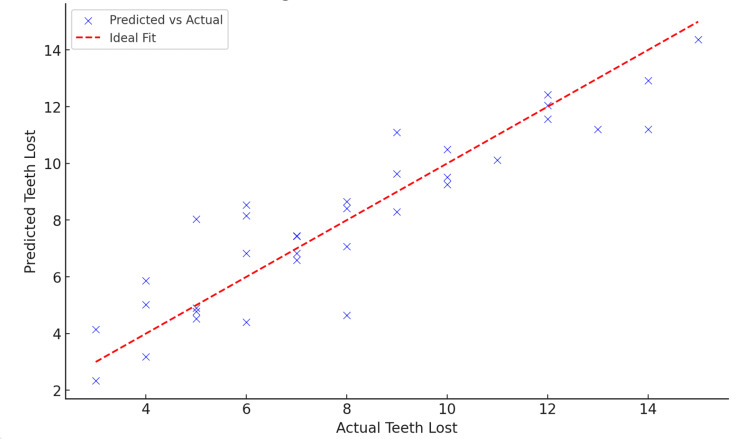
Multivariate regression: actual vs. predicted teeth loss

The multivariate regression analysis, depicted in Figure 7, illustrates the prediction of the number of teeth lost based on age, smoking status, diet quality, and mental health. A scatterplot demonstrates the model's predictive accuracy, with most data points closely aligning with the ideal line of fit, indicating a good model fit. Additionally, a bar graph of coefficients highlights the direction and magnitude of each predictor's effect on tooth loss. Age and diet quality emerged as the primary drivers of tooth loss in this analysis, consistent with existing literature on dental health. While smoking and mental health did not achieve statistical significance in this model, their potential influence may become more apparent with a larger sample size or the inclusion of additional covariates. The statistical model underscores the importance of focusing on modifiable factors, such as diet quality, in preventive dental health strategies. The graphical representation of these findings enhances understanding by emphasizing the relative impacts of the predictors, providing valuable insights for both academic research and clinical applications.

The R-squared value of 0.827 indicates that approximately 82.7% of the variability in tooth loss is explained by the predictors in the model, highlighting its strong explanatory power. The adjusted R-squared of 0.804 confirms a robust model fit while accounting for the number of predictors. Age showed a significant positive relationship (β = 0.2429, p < 0.001), indicating that older individuals are more likely to experience tooth loss. Diet quality demonstrated a significant negative relationship (β = −0.8506, p = 0.013), suggesting that better diet quality reduces the likelihood of tooth loss. Smoking (p = 0.694) did not show a statistically significant effect in this model. Similarly, mental health (p = 0.142) was not statistically significant, though its positive coefficient suggests a potential association with increased tooth loss, warranting further investigation with larger sample sizes or additional variables. These findings emphasize the importance of age and diet quality as key determinants of oral health while highlighting the need for future research into the roles of smoking and mental health.

**Figure 8:**
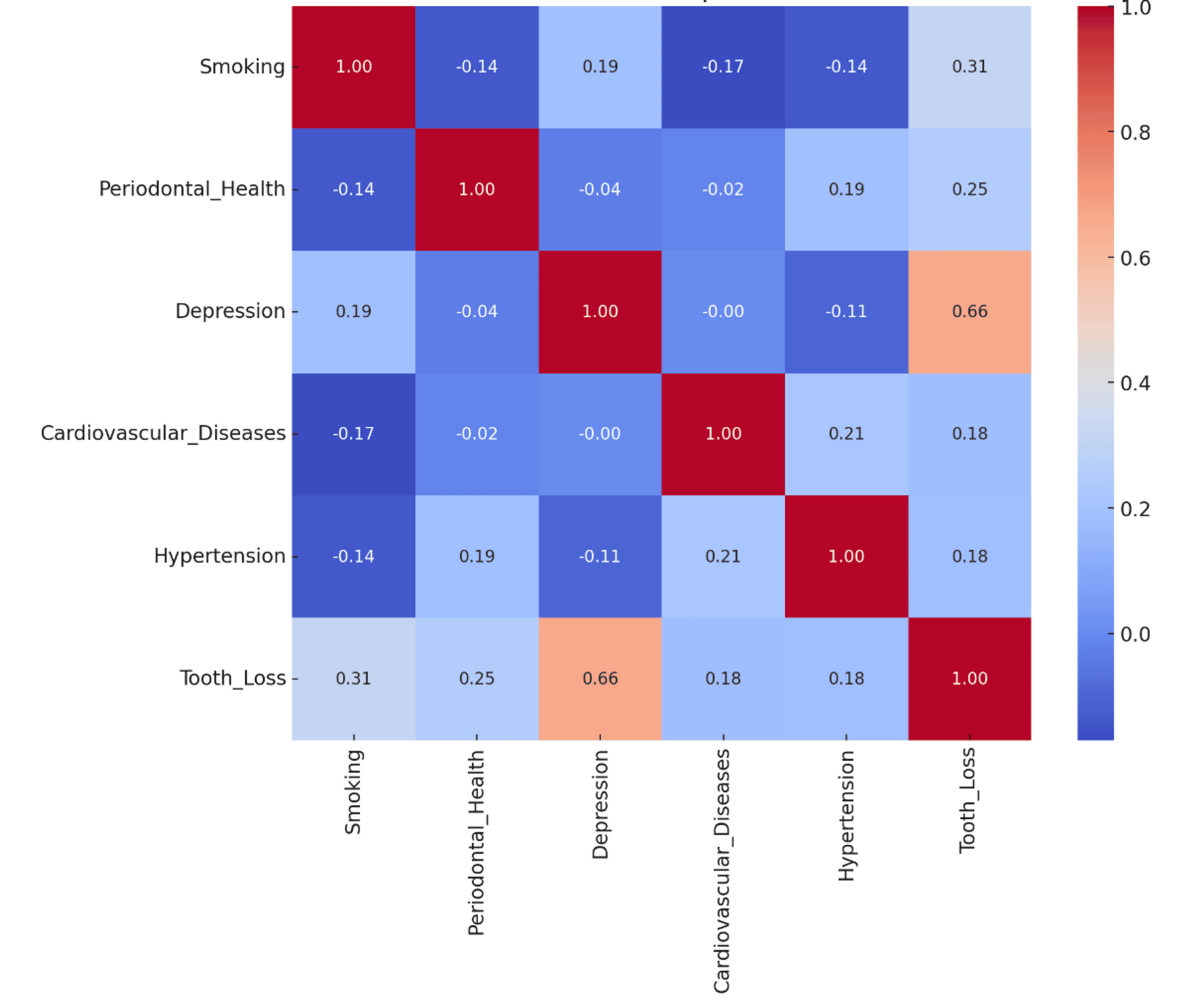
Matplotlib correlation heatmap of variables

The correlation heatmap in Figure 8 illustrates the associations between tooth loss of 35 participants over a 15-year period, with a total tooth loss of 321 teeth and five critical factors: smoking, periodontal health, depression, cardiovascular diseases, and hypertension. The R-squared = 64.9%, indicating that the selected predictors explain 64.9% of the variability in tooth loss. A high R-squared value reflects the strong collective influence of these factors, smoking, periodontal health, depression, cardiovascular diseases, and hypertension, on predicting tooth loss. The adjusted R-squared = 63.0% accounts for the number of predictors in the model, penalizing overfitting. The slight reduction from the R-squared value confirms that the model remains robust and reliable. The significant predictors (p < 0.05) indicate that all predictors were statistically significant, indicating that their impact on tooth loss is unlikely due to chance. This heatmap and its associated statistical analysis underscore the multifactorial nature of tooth loss and highlight the importance of considering these interrelated variables in clinical and public health strategies.

Depression has the strongest impact among the predictors, indicating its substantial role in tooth loss (r = 0.66). It highlights the critical connection between mental health and oral health. Smoking (r = 0.31) is the second most influential factor, emphasizing its significant role in predicted tooth loss. Periodontal health (r = 0.25) demonstrates a positive association, with poor periodontal health correlating with increased tooth loss. Cardiovascular diseases (r = 0.18) also contribute significantly to tooth loss, possibly due to shared inflammatory pathways. Similarly, hypertension (r = 0.18), though having a smaller effect size, remains a significant predictor of tooth loss. All variables exhibit moderate to high positive correlations, supporting their inclusion in the regression model. The high correlation between smoking, depression, and periodontal health demonstrates their strong individual associations with tooth loss. Together, these predictors significantly influence tooth loss, underscoring their importance in understanding the multifactorial nature of oral and systemic health.

Based on the multivariate regression analysis, the findings confirm a strong relationship between tooth loss and systemic health conditions, particularly smoking, periodontal health, depression, cardiovascular diseases, and hypertension. Each of these factors independently and collectively contributes significantly to the prediction of tooth loss, reinforcing its role as a potential biomarker for overall health. Smoking emerges as the significant predictor of tooth loss, reflecting its harmful effects on periodontal tissues and its role in promoting systemic inflammation. Poor periodontal health directly correlates with tooth loss, emphasizing its critical role as a local driver of systemic inflammation. Depression significantly impacts oral health by contributing to poor self-care, increased inflammation, and the neglect of routine dental visits. Cardiovascular conditions and hypertension further contribute to tooth loss through shared inflammatory pathways and vascular health deterioration. These findings highlight the interconnected nature of oral and systemic health and the importance of addressing these factors in a holistic healthcare approach.

**Figure 9:**
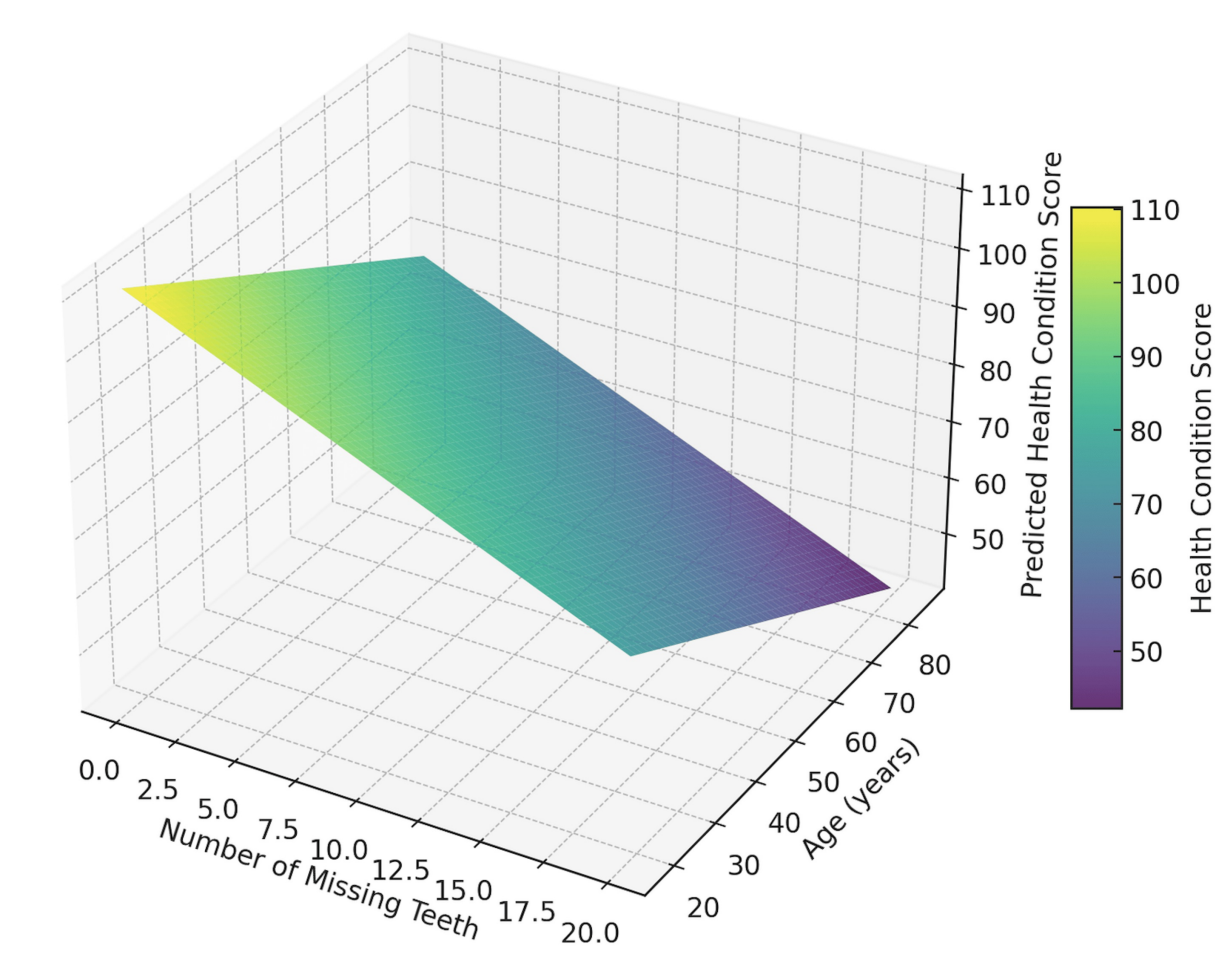
Predictive model of health status based on tooth loss and age

The predicted health condition based on tooth loss and age visualization, shown in Figure 9, is a 3D representation of the PHCSs derived from the number of missing teeth and age of 35 patients aged 36 to 70 years studied over a 15-year period with a total tooth loss of 321 teeth. The model represents the number of missing teeth, age, and the PHCS with a color gradient indicating health condition scores, where darker colors correspond to poorer health outcomes. This visualization highlights a clear trend: health condition scores decline progressively as age increases, and the number of missing teeth rises. This graphical representation provides a compelling depiction of the compounded impact of tooth loss and aging on systemic health, emphasizing the importance of preventive oral care and early interventions to mitigate broader health risks.

The equation derived from the simulated data to calculate the PHCS (denoted as y₁) is y₁ = mx + b = -1.95x + 78.12, where the slope m = -1.95 indicates that the health condition score decreases by 1.95 points for every additional missing tooth. The predictive model simulation used the formula y = mx + b. The Y variable represents the health condition score, a composite numerical representation of an individual's overall health status or severity index. The X variable represents the number of missing teeth. The m is the slope, which quantifies the change in the health condition score for every additional missing tooth. The b value is the intercept, representing the baseline health score when no teeth are missing (x = 0). After conducting predictive data simulations, the slope m = -1.95 consistently reflects the decrease in the health condition score for every additional missing tooth. The steeper negative slope underscores the strong relationship between tooth loss and health decline, emphasizing the significant impact of oral health on systemic well-being. This model highlights the importance of tooth retention in maintaining better health outcomes.

Based on the results from the linear regression model in this study, the formula to calculate the PHCS (denoted as y₂) was refined to incorporate the number of missing teeth and age. The predictive formula was expanded to y₂ = 0.0698x₁ + 0.0174x₂ + 75.256 = 0.0698 × number of teeth missing + 0.0174 × age + 75.256. The 0.0698 number is the coefficient for the number of missing teeth (x₁), indicating a positive impact on the health score; 0.0174 is the coefficient for age (x₂), also reflecting a positive impact on the health score; and the 75.256 is the intercept, representing the baseline health score when all predictors (number of missing teeth and age) are 0. It can be interpreted that for each additional missing tooth (x₁), the health score increases by 0.0698 points. For each additional year of age (x₂), the health score increases by 0.0174 points, while the intercept (75.256) represents the initial health score when the number of missing teeth and age is zero. This predictive formula highlights the combined influence of tooth loss and age on health scores, providing a more detailed framework for assessing health conditions.

The table of correlation coefficients (r) is a correlation matrix that provides the relationships between variables and presents a statistical breakdown with key observations, as represented in Table 3. The table data shows that tooth loss and depression show the strongest positive correlation (r = 0.6607), indicating a significant association. Smoking and tooth loss also exhibit a positive correlation (r = 0.3127), reflecting a relationship between smoking and the likelihood of tooth loss. Periodontal health and tooth loss are moderately correlated (r = 0.2480), highlighting the role of periodontal health in tooth loss. These values indicate varying strengths of relationships between the factors and tooth loss, emphasizing the multifactorial nature of the condition.

**Table 3:** Correlation coefficients

Conditions	Smoking	Periodontal health	Depression	Cardiovascular diseases	Hypertension	Tooth loss
Smoking	1	-0.1364	0.1908	-0.1702	-0.1362	0.3127
Periodontal health	-0.1364	1	-0.0366	-0.0176	0.1908	0.248
Depression	0.1908	-0.0366	1	-0.0003	-0.1061	0.6607
Cardiovascular diseases	-0.1702	-0.0176	-0.0003	1	0.2143	0.1783
Hypertension	-0.1362	0.1908	-0.1061	0.2143	1	0.1829
Tooth loss	0.3127	0.248	0.6607	0.1783	0.1829	1

The R-squared results indicate that the model explains 64.9% of the variance in tooth loss, demonstrating a strong predictive capacity. The adjusted R-squared, which accounts for the number of predictors, shows that the model explains 63.0% of the variance. Significant predictors are smoking with r = 0.3127, p < 0.001, periodontal health with r = 0.248, p < 0.001, depression with r = 0.6607, p < 0.001, cardiovascular diseases with r = 0.1783, p = 0.003, and hypertension with r = 0.1829, p = 0.003. All these factors contribute statistically significantly to tooth loss, highlighting the multifactorial nature of the condition. These results emphasize the importance of addressing behavioral, systemic, and psychological factors in strategies to reduce tooth loss.

Based on the multivariate regression analysis, the predictive equation for the PHCS (denoted as y) is as follows: health score = 100.73 − 0.80 × (teeth missing) − 0.42 × (age). The intercept of 100.73 represents the baseline health score when both the number of teeth missing and age are zero. The teeth missing coefficient (-0.80) indicates that each additional tooth lost reduces the health score by 0.80 units, assuming age remains constant. The age coefficient (-0.42) implies that each additional year of age reduces the health score by 0.42 units, assuming the number of teeth missing remains constant. Model fit of R-squared = 0.913 indicates that the model explains 91.3% of the variation in health scores, demonstrating strong predictive power. The adjusted R-squared of 0.911 is adjusted for the number of predictors, confirming the model's reliability. The F-statistic (508.2, p < 0.0001) shows that the model is statistically significant overall, meaning the predictors collectively strongly impact the health score. This model provides a robust framework for predicting health scores based on the number of teeth missing and age, emphasizing the significant impact of these factors on overall health.

This analysis indicates that tooth loss and age are significant predictors of health scores, with tooth loss exerting a slightly stronger impact on reducing health outcomes. The predictive equation derived can be utilized to estimate health scores based on these variables, providing valuable insights for health assessments. The correlation matrix highlights moderate associations between variables, such as tooth loss and depression (r = 0.6607), underscoring a notable and significant relationship. The Chi-squared statistic is 13.08, with a p-value of 0.9755 and a degree of freedom of 25. These results demonstrate a high p-value, suggesting no statistically significant relationships across the tested variables under the Chi-square analysis. This lack of significance in the Chi-square test may stem from weak associations within the underlying categorical data or constraints related to sample size. Despite this, the observed correlations between certain variables still suggest meaningful connections that warrant further exploration in larger, more comprehensive studies.

**Figure 10:**
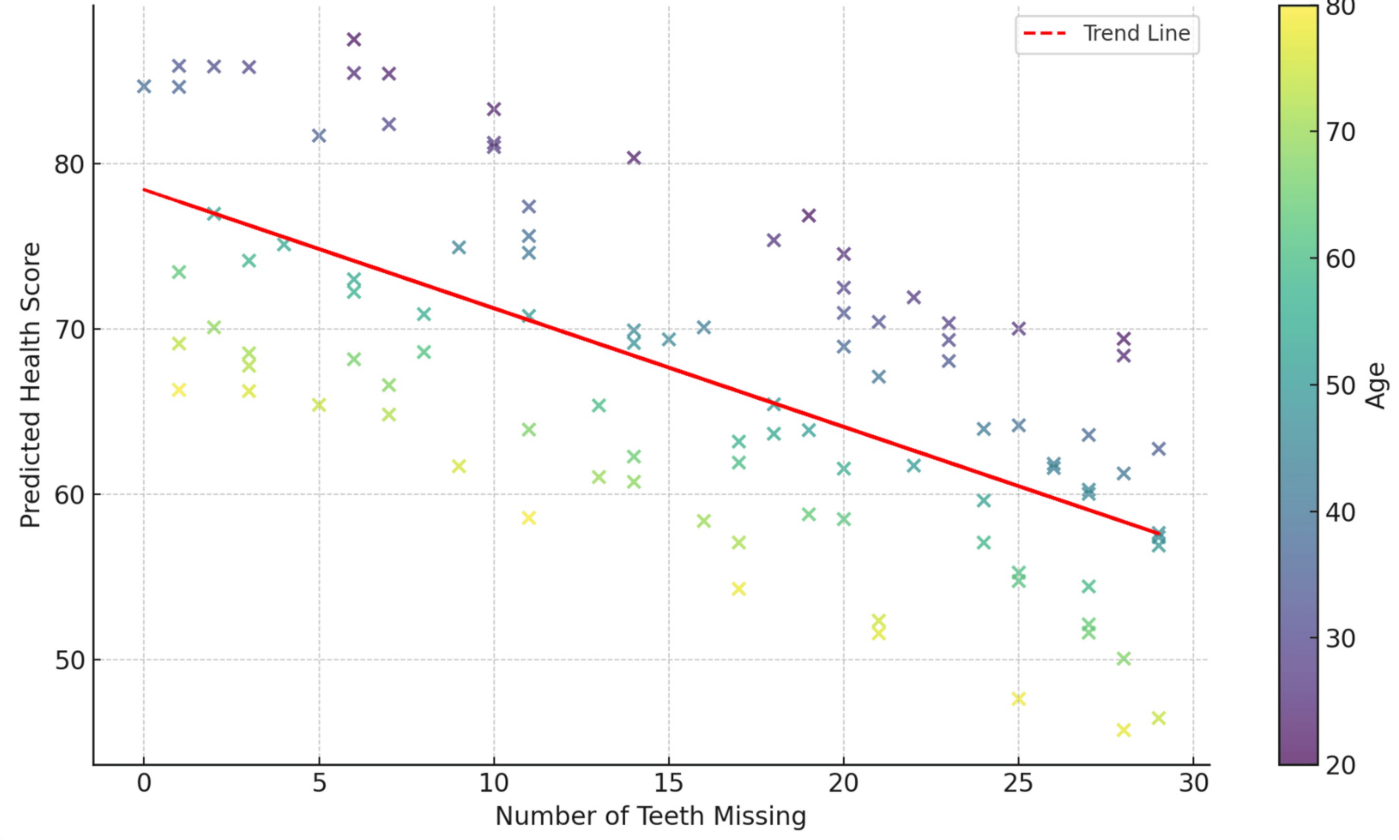
Health score prediction by teeth missing and age

Health score prediction by teeth missing and age scatter plot, presented in Figure 10, depicts the predictive equation representation for health scores based on the number of teeth missing and age of 35 patients aged 36 to 70 years studied over a 15-year analysis period with a total tooth loss of 321 teeth. The scatter plot reveals that health scores decrease as missing teeth and age increase. The red trend line illustrates the negative relationship between tooth loss and health scores, as predicted by the regression model. This visual representation underscores the interplay between these variables and reinforces the equation's capability to predict health outcomes accurately.

The final predictive equation was developed to provide a more accurate assessment of health scores by incorporating tooth loss and age as predictive factors. This equation calculates the PHCS (denoted as y₃), offering a practical tool for identifying patients who may benefit from further systemic health evaluation or preventive care interventions. The final predictive equation is as follows: y₃ = 100.73 - 0.80 × x₁ - 0.42 × x₂. In the equation y₃ = PHCS, x₁ = number of teeth missing, and x₂ = patient age in years. The key components of the equation are the intercept (100.73), which represents the baseline health score when both the number of missing teeth and age is zero; the coefficient for tooth loss (-0.80), indicating that each additional tooth lost reduces the health score by 0.80 units, holding age constant; and the coefficient for age (-0.42), meaning that each additional year of age reduces the health score by 0.42 units, holding the number of missing teeth constant. This equation highlights the combined influence of tooth loss and age on health outcomes, demonstrating its value as a quick biomarker or screening tool for healthcare providers.

Table 4 shows calculations using three different equations derived from this study's multivariate regression analysis, with the third equation being the most accurate for calculating the PHCS. The third equation (y3) is more accurate in calculating the PHCS based on tooth loss and age together. By using age and the number of missing teeth, we can confidently predict a patient's health score, reflecting their overall medical status and highlighting potential uncontrolled medical conditions that may have contributed to tooth loss.

**Table 4:** Predictive health condition score calculations

PHCS	A patient 65 years of age with one missing tooth
Y1	y1 = mx + b = -1.9*x + 78.12 = 76.223 = 76
y2	y2 = 0.0698*x1 + 0.0174*x2 + 75.256 = 77.085 = 77
y3	y3 = 100.73 - 0.80 * x1 - 0.42 * x2 = 72.93 = 73
PHCS	A patient 81 years of age with 16 missing teeth
y1	y1 = mx + b = -1.9*x + 78.12 = 47.72 = 48
y2	y2 = 0.0698*x1 + 0.0174*x2 + 75.256 = 77.7822 = 78
y3	y3 = 100.73 - 0.80 * x1 - 0.42 * x2 = 53.73 = 54

## Discussion

The findings of this study underscore the multifaceted relationship between oral health and systemic diseases [2,10]. Chronic periodontal infection, a leading cause of tooth loss in this study's analysis, was strongly associated with systemic conditions such as diabetes, cardiovascular disease, and respiratory illnesses, as confirmed by other studies [2,10]. The shared inflammatory pathways between periodontal disease and these systemic conditions can explain this association. The release of inflammatory mediators, such as cytokines and interleukins, from periodontal tissues into the bloodstream contributes to systemic inflammation, exacerbating underlying health issues [11]. These findings align with previous research, suggesting that periodontal inflammation is a biological link to systemic health deterioration [2,10,11].

The study also highlights the significant impact of behavioral and lifestyle factors on oral and systemic health. Smoking, poor diet, and psychological conditions such as depression were prevalent among the participants and contributed to both tooth loss and systemic health complications [2]. Smokers exhibited higher rates of severe periodontal disease, which significantly increased their risk for systemic conditions like atherosclerosis and respiratory infections. Similarly, patients with poor dietary habits and mental health issues often neglect oral hygiene, resulting in an increased risk of dental caries and periodontitis. These behaviors not only compromised oral health but also created a feedback loop in which systemic conditions further exacerbated oral health issues.

Psychological disorders such as depression and anxiety play a crucial role in the oral health of many patients. Neglect of oral hygiene, often observed in individuals suffering from depression, led to severe periodontal disease and subsequent tooth loss [12]. Additionally, stress-induced behaviors, such as bruxism, caused mechanical damage to teeth, further compounding oral health problems. The data also revealed that psychological distress was not only a precursor to poor oral health but also a consequence of systemic diseases exacerbated by tooth loss [2,13]. This bidirectional relationship between psychological health, oral health, and systemic diseases underscores the importance of addressing mental health as an integral part of comprehensive healthcare.

The study's findings suggest tooth loss can be a predictive biomarker for systemic health conditions. Individuals who experienced significant tooth loss due to periodontal disease or other factors exhibited higher incidences of systemic diseases. For instance, patients with more than five missing teeth were more likely to have diabetes and cardiovascular diseases. Tooth loss should be considered a marker of an individual's overall health status, reflecting underlying systemic conditions, lifestyle choices, and psychological factors. This observation aligns with growing evidence that oral health reflects systemic health, making it a valuable diagnostic tool for healthcare providers. Integrating oral health evaluations into routine medical check-ups could enhance early detection and management of systemic conditions.

This predictive health score model provides a quantitative framework for predicting health scores based on tooth loss and age. It highlights the negative impact of these variables on systemic health and could be valuable in clinical settings to assess risks associated with aging and dental health. The model's accuracy improves when age is incorporated alongside the number of missing teeth, compared to using tooth loss as a single factor. The score is a quick biomarker or screening tool to identify patients who may benefit from further systemic health evaluations or preventive care interventions. The model establishes correlations between oral health (tooth loss) and broader health outcomes, providing a quantitative method to explore this relationship. Interpretation is based on the number of missing teeth, reflecting oral health status and linked to systemic inflammation, periodontal disease, and other health issues. Additionally, older age negatively impacts systemic health due to its natural association with the progression of chronic diseases. While the scale is not absolute, it offers a relative indicator based on the study's parameters. Further validation with additional data could refine its predictive accuracy and broaden its clinical applications.

The limitations of the study are multifaceted. A cohort study is a longitudinal design that follows participants over a period of time. The advantages of a cohort study include its ability to follow participants with a common risk factor or characteristic over a long time, allowing for robust cause-and-effect conclusions. It also enables researchers to identify the timeline over which behaviors contribute to diseases. Additionally, retrospective cohort studies can save time since they use existing data. However, there are significant challenges associated with cohort studies. Participant dropout increases the risk of bias, and participants' behaviors may change over time. Managing and controlling a large number of confounding variables can be difficult, and some may go unaccounted for. The lack of randomization makes cohort studies less robust than randomized controlled trials. Moreover, retrospective cohort studies carry the additional risk of sampling bias and missing data. They are further weakened by the reliance on pre-existing data, which may be incomplete or of variable quality, potentially affecting the reliability of the study's findings.

As argued by some statisticians, one statistical limitation of the study is evaluating the OR instead of the RR [14]. The OR and RR measure the association between exposure and outcome, but their applications differ. Statisticians often argue that studying the risk of disease (RR) in cohort studies is more appropriate. In contrast, the OR, which measures the odds of an outcome occurring in an exposed group compared to the odds of occurring in a non-exposed group, is more commonly used in case-control studies. The distinction lies in their interpretation: the OR represents how likely an outcome is to occur in the exposed group than in the non-exposed group. At the same time, the RR measures how much greater the risk of a disease is in the exposed group than in the non-exposed group. Although the OR is a ratio of two odds and RR is a ratio of two probabilities, the overall interpretation is similar. Suppose either the OR or RR is greater than 1. In that case, it indicates that the chances of experiencing an event or exposure are higher in the treatment group compared to the control group.

In addition, the OR represents the ratio of the odds of an event occurring in one group to the odds of it occurring in another group. While the OR can be a valid measure of association, it carries the risk of exaggerating the strength of an association, particularly when the event is common. Although ORs can be challenging to interpret as a measure of association, their use should be limited to case-control studies and logistic regression analyses. Substituting ORs for RR is not always appropriate, as they measure different aspects of association [14]. The RR quantifies how much greater the risk of disease is in the exposed group than in the non-exposed group. In contrast, the OR describes the odds of exposure among those with a disease versus those without. For example, an OR of 14.1 means "the odds of having the condition are 14.1 times higher in the exposed group than in the non-exposed group." While both metrics can be used to interpret the relationship between exposure and disease, RR is generally preferred in cohort studies for its more direct interpretation of risk.

Another limitation of the study is its sample size, which might be too small to draw certain conclusions [15]. A third variable, potentially unaccounted for, could influence the observed relationships. Additionally, unmeasured third variables may obscure true relationships in the correlation results, making them difficult to interpret [16]. Moderate positive correlations in the correlation heatmap between predictors indicate potential multicollinearity risks, which can affect the accuracy of regression estimates. Furthermore, multiple factors could act as predictors, complicating the analysis. The scatterplot reveals that predictions deviate significantly from the actual values, as the points are not tightly clustered around the ideal fit line. This observation underscores the model's limited predictive capability.

Further research with larger sample sizes and refined measures of the predictors could enhance the robustness of the model, including additional factors associated with tooth loss, such as chronic pain in areas beyond the oral cavity (e.g., pain in distant body sites where severe tooth loss increases the odds of chronic pain in non-oral regions), long-term care needs, and mortality [17-19]. For instance, predictors like genetic predisposition to tooth loss may be significant but were not accounted for in this study analysis, potentially obscuring the results [20].

The implications of this study extend beyond individual patient care to broader public health strategies. Collaborative efforts between dental and medical professionals are essential for early diagnosis and comprehensive management of systemic diseases. Programs aimed at enhancing oral hygiene education, promoting smoking cessation, and providing mental health support could significantly reduce the burden of oral and systemic diseases. Future research should prioritize larger, longitudinal studies to further validate the use of tooth loss as a biomarker for systemic conditions. Additionally, investigating the genetic and molecular pathways that link oral and systemic health could offer deeper insights into preventive and therapeutic strategies. By acknowledging the interconnected nature of oral and systemic health, this study advocates for an integrated approach to healthcare, emphasizing prevention, early intervention, and interdisciplinary collaboration.

## Conclusions

This study demonstrates that chronic periodontal infection, age-related factors, smoking, poor diet, and psychological conditions, such as depression and bruxism, are significant contributors to tooth loss. The correlation between the number of teeth lost and systemic conditions, such as diabetes (r = 0.72, p < 0.01) and cardiovascular disease (r = 0.68, p < 0.01), was both strong and significant, indicating a predictive relationship. Kaplan-Meier survival analysis revealed that patients with severe periodontal disease experienced a significantly reduced survival time without systemic complications, with a median time to systemic disease onset of seven years compared to 12 years in patients with better oral health (p = 0.03). Logistic regression identified chronic periodontal infection as a primary predictor of systemic diseases, with an OR of 3.5 (95% CI: 2.2-5.4) for cardiovascular disease and 2.8 (95% CI: 1.9-4.3) for diabetes.

The study's findings underscore that tooth loss is not merely a localized dental issue but a robust indicator of broader health risks. Logistic regression identified significant predictors of tooth loss, including smoking (OR = 3.8, p < 0.01), severe periodontal infection (OR = 4.6, p < 0.01), and high BDI scores (OR = 2.9, p = 0.02). Predictors of systemic conditions included tooth loss (OR = 2.7, p = 0.03) and smoking (OR = 3.1, p = 0.02). Chi-square tests demonstrated significant associations between smoking and systemic conditions (χ² = 18.4, p < 0.01) and between tooth loss and cardiovascular disease (χ² = 12.7, p = 0.02). T-tests further quantified these relationships. Diabetic participants with severe periodontal infection exhibited significantly higher HbA1c levels compared to those without (t = 3.4, p = 0.01). Smokers experienced significantly greater tooth loss than non-smokers (t = 4.2, p < 0.01). The findings collectively emphasize the strong interplay between oral and systemic health.
